# The Experience of People With Urinary Incontinence Using Invasive Devices in Pelvic Floor Muscle Training: A Qualitative Study

**DOI:** 10.1002/pri.70071

**Published:** 2025-05-23

**Authors:** Benedetto Giardulli, Gaia Leuzzi, Ottavia Buccarella, Marco Testa, Simone Battista

**Affiliations:** ^1^ Department of Neurosciences, Rehabilitation, Ophthalmology, Genetics Maternal and Child Health University of Genoa Genoa Italy; ^2^ Department of Physical Education and Rehabilitation Experimental Anatomy Research Group (EXAN) Vrije Universiteit Brussel (VUB) Brussels Belgium; ^3^ School of Health and Society Centre for Human Movement and Rehabilitation University of Salford Salford UK

**Keywords:** pelvic health, qualitative research, rehabilitation, urinary incontinence

## Abstract

**Background and Purpose:**

Pelvic floor muscle training (PFMT) is a first‐line conservative treatment for urinary incontinence (UI), often involving invasive devices such as vaginal or anal biofeedback. However, these devices can cause discomfort, negatively impacting PFMT engagement. We aimed to explore the experiences of individuals with UI regarding the use of invasive devices during PFMT.

**Methods:**

A qualitative study was conducted using semi‐structured interviews. Participants were recruited through purposive sampling and interviews were conducted online. Data were analysed using Reflexive Thematic Analysis.

**Results:**

Fourteen participants (50% women; 50% men; age: 58 ± 17 years) who underwent PFMT involving invasive devices were interviewed. Four themes were generated: (1) ‘A Tool for Pelvic Floor Muscles: From Obligation to Acceptance’ as participants initially expressed discomfort and anxiety, which gradually evolved into acceptance. (2) ‘Building Trust Through Gradual Exposure and Tailored Guidance’ since supportive, patient‐centred communication and gradual exposure were key elements to alleviating discomfort. (3) ‘A Compact Trainer for PFMT’ since devices enhanced pelvic floor muscle awareness and self‐management through biofeedback, fostering independence and adherence. (4) ‘”Users” Needs: Privacy, Accessibility, Usability, and Autonomy’ emphasising barriers like lack of privacy, high costs, and need for user‐friendly designs to enhance accessibility and integration into daily life.

**Discussion:**

The study highlights the journey of individuals using invasive devices during PFMT: from discomfort to acceptance. Gradual introduction, empathetic communication, and tailored rehabilitation approaches by physiotherapists seemed to improve user engagement and adherence with the devices. Participants emphasised the need for accessible, user‐friendly devices to overcome logistical and psychological barriers.

**Abbreviations**
PFMT: Pelvic Floor Muscle Training
RTA: Reflexive Thematic Analysis
UI: Urinary Incontinence

## 1 Introduction

Urinary incontinence (UI) is a prevalent condition, with worldwide estimates suggesting that 25%–45% of women and 11%–34% of men report some degree of it (Buckley and Lapitan 2010). UI can impact the psychological dimension of people, hindering various aspects of their lives (National Institute for Health and Care Excellence (NICE) 2019). Evidence highlights how UI is strongly associated with overall poor levels of health‐related quality of life and reduced sexual well‐being, likely due to the incapacity of controlling urine loss in social and intimate contexts (Pizzol et al. 2021; Corrado et al. 2020; Lim et al. 2016). To improve UI symptoms, the first line of recommended conservative treatment is pelvic floor muscle training (PFMT) ((UK) NGA, 2021; Alouini et al. 2022), consisting of progressive exercises involving pelvic floor muscles aimed at maximising a person's functional capabilities (Cacciari et al. 2021).

In its early stages, PFMT often involves the adoption of devices, mainly endocavitary (i.e., vaginal or anal), with different purposes. One is to offer a facilitation to awareness of pelvic floor muscle contractions to exercise effectively through biofeedback, typically delivered via visual or auditory cues derived from pressure, ultrasound or electromyographic signals (Mazur‐Bialy et al. 2020). Another purpose is to offer resistance to voluntary contractions, thereby potentially enhancing muscle fibre strength. A third is to induce a local muscle effect (e.g., relaxation or strength improvement) through electrical stimulation (Bo et al. 2017). Examples of endocavitary devices used during PFMT are weight kegel balls, yoni eggs, vaginal or anal cones or dilators, vibrating devices, and biofeedback devices (Ong et al. 2015; Wang et al. 2023; Thomas et al. 2021). These devices have been shown to improve symptoms of urinary incontinence and, when present, sexual dysfunction (Bo et al. 2017). These devices aim to produce both physical effects and behavioural effects by increasing body awareness and encouraging active participation, supporting the view of PFMT as both a physical and behavioural therapy (Frawley et al. 2017; Giardulli et al. 2025).

However, using these devices may cause discomfort during PFMT (H. N. Lee et al. 2013). Specifically, the literature highlights that nudity and invasive physical contact with internal, intimate body parts, as well as manual techniques performed by the physiotherapist (e.g., digital vaginal or rectal examination), can lead participants to feel embarrassed and displeased, influenced by concerns about potential pain and cultural factors (Giardulli et al. 2024; Kondo et al. 1995; Fernandes et al. 2025). As a result, this discomfort may negatively affect adherence to PFMT (H. N. Lee et al. 2013). Moreover, these devices often require the user to be naked in supine, seated, or lateral decubitus positions, making it challenging to establish an appropriate setting for training (Bo et al. 2017).

Quantitative research provides conflicting evidence towards the added value of endocavitary devices during PFMT in people with UI. Systematic reviews have reported no differences or inconclusive results in terms of incontinence quality of life, or symptom cure or improvement, when investigating the added benefits of devices to PFMT in people with UI (Fernandes et al. 2025; Benedetto et al. 2023). However, in Italy, clinical guidelines for the conservative management of UI recommend using biofeedback and electrical stimulation—often delivered via endocavitary devices—as part of the physiotherapy intervention (Società Italiana di Urologia and European Association of Urology 2019). Therefore, exploring the experiences and perceptions of people with UI regarding invasive devices during PFMT could help to get valuable insights into how these devices are perceived and the emotions they evoke (Oben 2020). Qualitative research, by exploring the beliefs, experiences, and perspectives, can further enhance understanding of patients' experiences. This knowledge can guide physiotherapists in addressing users' discomfort and proposing strategies to overcome their engagement and satisfaction (Oben 2020; Maher and Dertadian 2018).

Hence, the following study aimed to understand the experience of using invasive devices, regardless of whether they offered only biofeedback or a local muscle effect, during PFMT in people with UI. By offering insights into users' emotional responses and engagement strategies, this study aims to inform more patient‐centred PFMT practices and contribute to improving treatment satisfaction.

## 2 Methods

### 2.1 Study Design

We conducted an interview qualitative study to explore the experience of invasive devices of people with UI who underwent PFMT. Ethical approval was obtained from the Ethics Committee for University Research (CERA) of the University of Genova, Italy (CERA2023/80; approval date: 31/10/2023). The study followed the ‘Declaration of Helsinki’ and is reported in line with the ‘Consolidated Criteria for Reporting Research for reporting qualitative studies’ (COREQ) (Booth et al. 2014).

### 2.2 Participants

Adult (+18) people with UI who underwent a PFMT programme involving an invasive device, such as anal or vaginal biofeedback, electrical stimulation, weight kegel balls, or cones, were recruited adopting a purposive sampling. This approach was chosen to ensure the inclusion of participants with direct and relevant experience related to the research question. Specifically, we aimed to include Italian‐speaking participants of different genders, age groups, sexual orientations, and experiences with different types of intracavitary devices; however, although diversity in age and gender was achieved, the final sample reflected heterosexual participants. To reach participants, different pelvic floor physiotherapists were contacted to share the opportunity to partake in the study with their patients, as well as aims, inclusion criteria and procedures. Once they received enough information on the study's purposes, patients were invited to contact BG if they were interested in partaking. Moreover, emails, social media and snowball sampling were used to reach as many participants as possible and maximise the diversity. Online interviews allowed us to reach more Italian regions from the north, mid and south. The informed consent form and the informative note were shared with participants to explain the objective and modality of the study. No restrictions were applied to gender and age. Participants could withdraw from the study at any moment without the need for an explanation.

### 2.3 Research Team

The research team was composed by B.G., G.L., O.B., M.T. and S.B. B.G. is a physiotherapist and PhD in Neurosciences. GL is a sports scientist and a joint PhD student in Neurosciences and Rehabilitation and Physical Education at the University of Genova (Genova, Italy) and the Vrije Universiteit Brussel (VUB, Belgium). O.B. is a physiotherapist with expertise in pelvic floor rehabilitation. M.T. is a physiotherapist, PhD in Rehabilitation Sciences and associate professor at the University of Genova (Genova, Italy). S.B. is a physiotherapist, PhD in ‘Neurosciences’ and ‘Medical Science’ and research fellow at the University of Salford (Salford, United Kingdom). B.G., S.B. and M.T. identify themselves as men; G.L. and O.B. identify as women. B.G., G.L. and S.B. are trained in conducting qualitative studies.

### 2.4 Data Collection Method

A team of physiotherapists (BG, SB, OB, MT) developed a semi‐structured interview guide with open questions (Table 1). No pilot testing was conducted. The interview guide was specific for the endocavitary devices, as the general expectations, beliefs and experience of PFMT, in the same population, have been explored in another study (Giardulli et al. 2024). As such, we did not seek to replicate those findings but provide a focused exploration of participants' perspectives on the use of invasive devices, considering that PFMT users reported a degree of discomfort also during vaginal examination and digital rectal palpation (Giardulli et al. 2025, 2024). Hence, the interviews aimed to capture the lived experience, including the thoughts and beliefs of participants about the use of endocavitary devices. Before the interview, sociodemographic data, such as age, gender, sexual orientation, educational attainment, employment, marital status and clinical conditions, were collected and registered on an Excel sheet. B.G. conducted one‐to‐one online interviews in Italian on Microsoft Teams to prevent the embarrassment of a group interview (i.e., focus groups) on intimate topics. Online interviews facilitated participation by removing the need for physical travel and allowing participants to remain in a comfortable environment (e.g., home) and fostering privacy when talking about intimate topics and experiences. Moreover, participants could choose to disable or turn off their webcam if it made them feel more at ease, preserving their visual privacy—important given the sensitivity of the topic.

**TABLE 1:** Interview guide.

Questions	Type	Dimension
1. How would you describe your experience using internal anal/vaginal devices during your rehabilitation process? Could you describe which internal devices you have used? 2. What role did the device play in your rehabilitation process?	Introductory and specific questions	Experience of using internal devices
3. Could you indicate three advantages and three disadvantages you experienced in using internal devices?	Specific question	Experience of using internal devices
4. What was your reaction when you were first proposed the use of internal anal/vaginal devices? Did you have any prejudices about it? How did you feel about the specific way these internal devices need to be used, considering they have to be inserted internally at the perineal level? How did you feel while using them? And afterwards?	Specific question	Experience of using internal devices
5. Based on your experience, what advice would you give to someone who is using internal devices for the first time? Would you recommend them?	Specific question	Suggestions
6. What suggestions would you give to those who develop these devices or to the physiotherapist who recommends them?	Question to sum up	Suggestions
7. Is there anything we have not mentioned that you would like to add?	Conclusive question	Conclusion

Thanks to Microsoft Teams, interviews were audio and video recorded to have an auto‐transcribed *verbatim* file. BG manually corrected and anonymised (i.e., P1) transcription files, respecting the privacy and integrity of each interviewee. After the correction, original transcriptions were deleted. Each participant received an anonymous code based on their interview order, age and gender (e.g., P6, 55y, Woman). None of the participants had met the interviewer before.

### 2.5 Data Analysis

A descriptive analysis was adopted for sociodemographic data. Transcribed data were analysed by adopting the ‘Reflexive Thematic Analysis’ (RTA) by Braun & Clarke (Braun and Clarke 2021a) to get patterns of meaning among the dataset. RTA belongs to the ‘Big Q’ qualitative paradigm and allows for identifying common patterns of meaning in the data. Moreover, it does not adhere to ‘small Q’ strategies such as minimising bias, coding accuracy and using other strategies like data saturation and member checking to increase trustworthiness (Braun and Clarke 2021b). Hence, the sample size was decided following the ‘information power’ model, which reports that the number of participants depends on the study aim, sample specificity, theoretical background, quality of dialogue, and the analysis method (Malterud et al. 2016). Given the narrow aim of the study, the sample specificity, the theoretical perspectives of the study, the expertise of the researcher conducting the interviews and in the pelvic clinical field (B.G.), and the purposive sample selection, we estimated a sample of participants between 12 and 16. Specific attention was given to diversify the group by considering participants' gender and age. The specific methodology of RTA followed for this study is represented in Table 2. Data codification was inductive to allow the researchers to catch the richness and variability of data without imposing any framework. The objective was to identify reflexed shared meanings among the dataset, adopting an experiential qualitative theory and a realistic framework (Braun and Clarke 2021a, 2021b). The use of field notes, like memos and diary, during interviews was allowed to promote reflexivity. Data codification was semantic, allowing researchers to stay on the explicit or surface of the meaning of data. When possible, researchers also adopted a latent approach, going beyond the surface data content to interpret underlying meaning, assumption, and ideas. The participants' and researchers' original language (Italian) was used to develop codes and themes, which were then translated into English by B.G. for publication (Yunus et al. 2022). To ensure conceptual accuracy and preserve the transferability of research findings, we adopted the rechecking process (Yunus et al. 2022). B.G. and S.B. are proficient in the English language, and they compared the translation with the original data, checking that the original meaning was not lost. Moreover, the entire team reviewed and agreed on the translations.

**TABLE 2:** Six steps of the ‘reflexive thematic analysis’.

Phases	Process	Authors' involvement	Authors' actions
(1) Data familiarisation	Authors immerged themselves in the data to understand the depth and breadth of the content.	Authors became familiar with the data in this phase.	Reading and re‐reading data set; Listening to the audio recordings; Taking notes; Marking transcripts sections relevant to the research question.
(2) Coding	Two authors generated codes to organise the dataset, giving full and equal attention to all data items.	B.G. and G.L. read individually the transcripts again and developed the recurring ideas into initial codes at the semantic level. Afterwards, all relevant extracts were coded.	Peer debriefing: Memos were shared during research meetings for reflexive thoughts; Labelling and organising data items into meaningful groups.
(3) Generating initial themes	Two authors generated initial themes by sorting codes and identifying the meaning of and relationships between codes.	B.G. and G.L. reviewed the codes and looked for broader patterns of meaning which were developed into preliminary themes.	Diagramming or mapping to make sense of theme connections; Writing themes and their defining properties.
(4) Reviewing and refining themes	The authors reviewed the initial themes, reworking or eliminating some until finding a set of themes fitting the dataset.	B.G. and G.L. discussed and revised the preliminary themes to ensure they represented the codes and the overall patterns identified in the data.	Ensuring there is enough data to support a theme; Re‐working and refining codes and themes.
(5) Defining and naming themes	Authors developed themes' names and refined them as they could tell a ‘story’.	B.G., G.L., and S.B. refined the definition and name of each theme and wrote a preliminary report outlining each theme. Themes were reviewed to ensure the themes represented participants' experiences and perspectives.	Peer debriefing and team consensus on themes; Cycling the data and the identified themes in order to organise the story.
(6) Producing the report	All authors produced the final report and refined the themes if necessary.	All authors gave feedback and contributed to refine the preliminary analysis.	Writing the final report; Report on reasons for theoretical, methodological, and analytical choices.

## 3 Results

From June to October 2024 14 Italian people with UI agreed to partake in the study (Age [Mean ± Standard Deviation]: 58 ± 17 years, 50% Women, *N* = 7; 50% Men, *N* = 7). All participants were heterosexual and white Caucasians. No repeated interviews were conducted. Each interview lasted in mean 12 (SD = 4) minutes. More details are reported in Table 3.

**TABLE 3:** Participants' characteristics.

Participant	Gender	Sexual orientation	Age	Educational attainment	Employment	Marital status	Clinical condition
P1	W	Heterosexual	29	Master's degree	Employee	Unmarried	UI, hypothyroidism
P2	W	Heterosexual	27	Master's degree	NDT	Unmarried	UI, vulvodynia
P3	M	Heterosexual	75	Master's degree	Retired	Married	UI PP
P4	M	Heterosexual	69	Master's degree	Physician	Married	UI PP
P5	W	Heterosexual	62	High school	Retired	Married	UI, uterine and bladder prolapse
P6	W	Heterosexual	45	Master's degree	Psychologist	Married	UI
P7	W	Heterosexual	53	Bachelor's degree	Physiotherapist	Married	UI, Hodgkin lymphoma, cardiopathy, asthma
P8	W	Heterosexual	36	Master's degree	Physiotherapist	Married	UI
P9	W	Heterosexual	61	High school	Unemployed	Married	UI, hypercholesterolaemia
P10	M	Heterosexual	69	Secondary school	Entrepreneur	Married	UI PP
P11	M	Heterosexual	76	Master's degree	Physician	Divorced	UI PP
P12	M	Heterosexual	76	High school	Retired	Married	UI PP
P13	M	Heterosexual	66	Bachelor's degree	Physiotherapist	Married	UI PP, hypertension, blind person
P14	M	Heterosexual	77	Master's degree	Retired	Married	UI PP

Abbreviations: M, man; N, number; NDT, neurodevelopmental disorders therapist; PP, post‐prostatectomy; UI, urinary incontinence; W, woman.

### 3.1 Reflexive Thematic Analysis

From the analysis of the interviews, a total of four themes were created: (1) ‘A Tool for Pelvic Floor Muscles: from Obligation to Acceptance’, (2) ‘Building Trust Through Gradual Exposure and Tailored Guidance’, (3) ‘A Compact Trainer for PFMT’, (4) ‘”Users” Needs: Privacy, Accessibility, Usability, and Autonomy’. Codes, and relative quotations, that led to the generation of these themes are reported in Tables 4, 5, 6 to 7.

**TABLE 4:** Illustrative data extracts for Theme 1: ‘A Tool for Pelvic Floor Muscles: from Obligation to Acceptance’.

Codes defined by researchers	Example of quotes extracted from the interviews
Accepting for a good reason	‘(The device) initially, I was a bit puzzled. But in the end, I accepted it because I knew I had to solve this problem, you know’.—Participant 3, 75 yo, man
	‘When the doctor suggested it, well, it wasn't easy for me … It created a bit of a problem. But, you see, the need to fix it helps you overcome everything’—Participant 10, 69 yo, man
	‘The first time, it wasn't exactly pleasant because, you know … but after the surgery, I didn't have those hesitations or issues anymore. The problem was so obvious and serious that I was willing to do anything to solve it’—Participant 12, 76 yo, man
	‘I was resigned and convinced that I needed to solve the problem because incontinence is inconvenient … it's uncomfortable and unpleasant. So, I would face any situation to resolve it.’—Participant 10, 69 yo, man
	‘These are things a patient must accept … I wouldn't say accept passively, but accept with trust … not passively or submissively, this whole matter …’—Participant 14, 77 yo, man
Experiencing discomfort	‘My first reaction was … fear. But afterwards, using these devices made me feel better, I mean, they do make you feel good’.—Participant 2, 27 yo, woman
	‘Initially, there was a bit of … how should I put it? A bit of anxiety because I wasn't … I'm not used to these things; so I started off feeling a bit like that …’—Participant 9, 61 yo, woman
	‘I'm calm during use, but sometimes it can trigger a bit of anxiety or involuntary tension, like… you're afraid of something, even something trivial … I don't know, like wetting the treatment table …’—Participant 1, 29 yo, woman
	‘Even a small sound, like a knock at the door, is really disruptive. It causes more agitation, so you need to find a quiet moment, even if it's just 10 minutes, to use them’.—Participant 2, 27 yo, woman
	‘There's the inconvenience of being … let's say, naked, in front of the person performing this technique …’—Participant 4, 69 yo, man
	‘The first thing I thought was “oh God”, what am I doing? If I told someone, they'd think, “What on earth is this person up to?”’—Participant 2, 27 yo, woman
	‘She explained it to me. Well, you know, at my age, you still have some prejudices, partly because your sex life slows down a lot at this age. We're not used to all these things that younger people might use all the time …’—Participant 9, 61 yo, woman
Something unnatural	‘At first, it felt strange, you know, because I'd never explored that part of myself with something external before … not with, well, an inanimate object, so it was a strange sensation’.—Participant 2, 27 yo, woman
	‘I was okay leaving it aside … maybe it was my prejudice that it was something too invasive, or that it might make me think my problem was more serious and harder to overcome’.—Participant 6, 45 yo, woman
	‘(Speaking about the device being invasive and artificial) Well, yes, a little bit, yes, a little bit’—Participant 9, 61 yo, woman
	‘The initial impact is a bit painful, I mean, it does feel a bit invasive. And I don't know, maybe it’s the idea that it’s not something natural, you know? It feels like an artificial intervention’—Participant 6, 45 yo, woman
	‘I had this prejudice that introducing this machine meant I needed something extra because I wasn't capable on my own. Also, I saw it as invasive and unnatural’—Participant 6, 45 yo, woman
No discomfort	‘It wasn't something that embarrassed or worried me, you know’.—Participant 1, 29 yo, woman
	‘I got used to it, and it's not such an uncomfortable thing, right? Absolutely … it was more the thought, the initial impact, but then it's not something that causes problems … no discomfort …’—Participant 10, 69 yo, man
	‘A disadvantage … I don't see one, I don't see one because I have a good relationship with my body’.—Participant 5, 62 yo, woman

**TABLE 5:** Illustrative data extracts for Theme 2: ‘Building Trust Through Gradual Exposure and Tailored Guidance’.

Codes defined by researchers	Example of quotes extracted from the interviews
Gradual approach	‘I would recommend starting very gradually, especially because it's a part of the body people tend to ignore or avoid thinking about. So, starting gradually and having a very relaxing environment is important’.—Participant 2, 27 yo, woman
	‘Perhaps some people might need to be guided through it, but it's definitely something that helps a lot, so it's worth being supported to take up this type of activity’.—Participant 5, 62 yo, woman
	‘It felt like, at least in my case, I needed to prepare myself for the idea, try the experience first, see how it went, and then realise that I could try something more’.—Participant 6, 45 yo, woman
Trust in physiotherapist	‘I would have preferred more questions, you know, for them to ask more. They did it naturally, of course, but there's only half an hour, and you need to wake up, get dressed, and then there's the next patient. And then there's the machine, which, I don't know, is about finding a way to feel comfortable despite all these circumstances. So, as much as you want to build a friendly relationship, it can't always fully happen …’—Participant 3, 75 yo, man
	‘Talk about it, suggest it calmly, and let the person build confidence with it over time’.—Participant 5, 62 yo, woman
	‘They also took measures like covering me slightly and explaining in detail what they were about to do. They used all the appropriate techniques to make sure inserting the probe wasn't distressing. So, they focused on explaining and using the right products for the electrostimulation’.—Participant 13, 66 yo, man
	‘I approached it very calmly because I trusted my therapist’.—Participant 1, 29 yo, woman
	‘I quickly got to know my therapist and understood that they were advising me for my own good. So, I didn't feel stressed or like I was being forced into something against my nature.’—Participant 14, 77 yo, man
The role of guidance and education	‘Take it very gradually, providing explanations. Show the device, demonstrate how to use it, and explain what it’s for ....’—Participant 2, 27 yo, woman
	‘Do the exercises together the first time, like using a cycle of machines with the patient. Emphasise staying focused—for example, don't use it while looking at your phone or reading because the time will pass, and you'll get distracted’.—Participant 8, 36 yo, woman
	‘My advice is for the physiotherapist to provide good psychological preparation by explaining what it is, what it involves, and especially how to use it and the specific indications’.—Participant 12, 76 yo, man
	‘I would recommend them, but not for independent use, obviously. I'd advise strict monitoring and guidance. In my opinion, monitoring is essential—it completely changes not only the results but also how useful and effective the support is’.—Participant 8, 36 yo, woman
	‘I would definitely recommend it … but with guidance from a good pelvic physiotherapist’.—Participant 5, 62 yo, woman
	‘Doing it with the physiotherapist the first few times was an advantage because they gave clear guidance and instructions …’—Participant 12, 76 yo, man
Tailored approach	‘The foundation should be starting with the individual—understanding their timing and approach—and then introducing the device later. I Think starting right away with it isn't ideal …’—Participant 6, 45 yo, woman
	‘Another thing I'd like is for the therapist to adjust their approach depending on the person in front of them, adapting to their specific issues’.—Participant 3, 75 yo, man

**TABLE 6:** Illustrative data extracts for Theme 3: ‘A Compact Trainer for PFMT’.

Codes defined by researchers	Example of quotes extracted from the interviews
Active use	‘I think being an active part of rehabilitation really makes a difference. It makes you feel less useless during these times’.—Participant 2, 27 yo, woman
	‘It's about paying attention to what's happening, like the contraction. Because if it's something that also works passively, without our input, a person might not pay attention and won't engage with the exercise … it's about staying focused and not getting distracted’.—Participant 8, 36 yo, woman
	‘There are things I also need to do alongside the machine, like paying attention to the timing and other details’.—Participant 6, 45 yo, woman
Self‐management	‘The sequence of exercises and everything is preset, so no collaboration is needed’.—Participant 8, 36 yo, woman
	‘The setup was predetermined, so timing, frequencies, pauses, and impulse rates were already set. It was simple and easy for me to use, and as a blind person, I didn't encounter any difficulties’.—Participant 13, 66 yo, man
	‘I felt happy being able to evaluate my improvements over time. The immediate effect was relaxation—after just one session, I could feel my muscles were more relaxed, which was very positive’.—Participant 1, 29 yo, woman
	‘I think having it available for a ‘refresh’ of the contraction perception would be ideal’.—Participant 7, 53 yo, woman
Difficulty in adhering to PFMT	‘It definitely played an important role because it “forced” me to do the exercises. I find it difficult to do them every day on my own’.—Participant 7, 53 yo, woman
	‘Balancing it with other commitments was challenging … even that half‐hour I had to carve out felt like a sacrifice, but it was important’.—Participant 6, 45 yo, woman
	‘The need for continuity can also be a drawback’.—Participant 8, 36 yo, woman
	‘The advantage is knowing exactly how long it takes. If the programme is 20 min, it's 20 min, no more. So, it's easy to plan for’.—Participant 8, 36 yo, woman
	‘I think it also played a role in psychological relief. It became tyring—both finding the time and then having to make that time for myself’.—Participant 6, 45 yo, woman
Integrating with PFMT	‘(My advice is) to prescribe these devices, but also the exercises to do independently at home …’—Participant 11, 76 yo, man
	‘Initially, my situation was severe, but by using the probe, I gradually saw improvements. They were slow—it took six or 7 months—but I noticed progress, always accompanied by the exercises …’—Participant 12, 76 yo, man
	‘The experience was positive, showing improvement. I saw progress in conjunction with doing pelvic exercises myself … the device was an excellent support’.—Participant 13, 66 yo, man
	‘It's designed to help you perform your exercises independently. It's a tool to reach a level where you can manage your pelvic floor on your own’.—Participant 5, 62 yo, woman—Participant 5, 62 yo, woman
Improvements	‘Using it was good because I could see the improvement, which was confirmed by the therapist. The contractions kept improving more and more, day after day’.—Participant 4, 69 yo, man
	‘In my opinion, it was indispensable for achieving the result’.—Participant 5, 62 yo, woman
	‘The most important advantage was being able to perceive what my pelvic floor was doing and the benefits it brought, including improvements in my sexual life’.—Participant 1, 29 yo, woman
Contraction awareness	‘They help you get feedback on what your body is doing … you don't always feel the movements you're making, but with these tools, I could understand and perceive how much I was squeezing, how much I could hold. So, feedback is definitely the most fitting word’—Participant 1, 29 yo, woman
	‘The benefit is seeing—because it's a biofeedback technique—seeing visually how my contraction was progressing and following it step by step. It motivated me to do it properly because I could see it happening’—Participant 4, 69 yo, man
	‘Because moving internal muscles isn't as straightforward as moving your arms or legs, so this was fundamental’.—Participant 5, 62 yo, woman
	‘It supported my contraction, giving me information that helped me better understand what kind of movement I needed to do, or rather its intensity and duration’—Participant 8, 36 yo, woman

**TABLE 7:** Illustrative data extracts for Theme 4: ‘”Users” Needs: Privacy, Accessibility, Usability, and Autonomy’.

Codes defined by researchers	Example of quotes extracted from the interviews
Appropriate setting	‘A drawback is having to find a moment for yourself to dedicate to (rehabilitation)’.—Participant 1, 29 yo, woman
	‘You need a quiet place to do them, so it requires some preparation. If you're feeling stressed, your mind races, and it could cause anxiety in certain individuals’.—Participant 2, 27 yo, woman
	‘So, this thing about creating the setting, let's say, is more of a negative aspect’.—Participant 8, 36 yo, woman
	‘The drawback, so to speak, is that you need to lie down and not have any clothing on, etc. So, from a practical perspective, you need to create a specific moment to use it, much more structured than just doing exercises’.—Participant 8, 36 yo, woman
Discretion	‘I needed to fly there, and I had to carry the case, which took up a lot of luggage space. So, the disadvantage is its lack of practicality if you need to travel—you have to bring the kit with you’.—Participant 6, 45 yo, woman
	‘The fact is that it’s not something you can just put in a bedside drawer. Aesthetically, it's not very easy to manage’—Participant 7, 53 yo, woman
Accessibility	‘The cost—some of them are 80 or 90 euros. Especially for those who undergo a lot of pelvic floor therapies, it's a helpful tool but an expensive one …’—Participant 2, 27 yo, woman
	‘Making them tax‐deductible could help. I had to buy mine from a Spanish website because I couldn't find it in Italy’.—Participant 1, 29 yo, woman
	‘Ease of use. I had trouble with some devices, for instance, setting the heat… It should be something that people can manage themselves, automatically’.—Participant 2, 27 yo, woman
	‘Some of these devices aren't accessible because they can only be used by certain professionals. The ones you can use on your own don't, in my opinion, provide the same benefits’—Participant 2, 27 yo, woman
	‘Many people assume you don't need lubricant, but I think this should be made clear. I read about it or was told, but the availability to adapt or make changes is crucial if someone isn't ready yet’—Participant 2, 27 yo, woman
	‘One piece of advice I'd give is to use lubricants properly. At my age, we start experiencing dryness and such things …’—Participant 9, 61 yo, woman
	‘There are very few of us, I hope, with this problem (blindness) … but it would be helpful to integrate a small voice synthesis for greater independence’—Participant 13, 66 yo, man

#### 3.1.1 Theme 1: ‘A Tool for Pelvic Floor Muscles: From Obligation to Acceptance’

This theme was generated by exploring participants' emotional responses to invasive devices, focussing on their initial reactions and journey over time (Table 4).

Participants expressed mixed feelings toward the use of invasive devices. Initially, many experienced anxiety and discomfort about the idea of using an endocavitary device, particularly in the context of being undressed in front of a physiotherapist. Some participants even described the use of endocavitary devices as ‘something not natural’ due to its invasiveness. However, over time, some participants changed their minds and became comfortable with the device. Specifically, they reported an initial prejudice that after making the experience of the devices disappeared.

I had this prejudice that introducing this machine meant I needed something extra because I wasn’t capable on my own. Also, I saw it as invasive and unnatural—Participant 6, 45 yo, woman

Men were often reluctant towards endocavitary devices but viewed them, or were guided to see them, as a necessary step for relieving their symptoms. Therefore, men approached the devices as a required and discomforting but worthwhile obligation.

I was resigned and convinced that I needed to solve the problem because incontinence is inconvenient… it’s uncomfortable and unpleasant. So, I would face any situation to resolve it.—Participant 10, 69 yo, man

Conversely, a minority of participants reported no issues using invasive devices from the beginning, highlighting how having a good relationship with your own body could help.

A disadvantage… I don’t see one, I don’t see one because I have a good relationship with my body.—Participant 5, 62 yo, woman

#### 3.1.2 Theme 2: ‘Building Trust Through Gradual Exposure and Tailored Guidance’

This theme was generated by exploring the factors that helped participants approach invasive devices for the first time and overcome initial discomforts and barriers (Table 5).

Participants emphasised how being gradually introduced and prepared for the use of invasive devices helped them. This step‐by‐step approach involved physiotherapists presenting the idea repeatedly but with kindness and discretion, ensuring patients felt supported rather than pressured. Positive communication, characterised by an empathetic and patient‐centred narrative, fostered confidence and comfort. This helped participants feel safe and guided through what could be an intimidating experience.

It felt like, at least in my case, I needed to prepare myself for the idea, try the experience first, see how it went, and then realise that I could try something more.—Participant 6, 45 yo, woman

Moreover, a deep sense of trust in the physiotherapist was necessary in alleviating the discomfort associated with endocavitary devices. This trust allowed participants to view the device as a valuable tool in their rehabilitation process rather than a source of distress.

I quickly got to know my therapist and understood that they were advising me for my own good. So, I didn’t feel stressed or like I was being forced into something against my nature.—Participant 14, 77 yo, man

Finally, participants highlighted the importance of being educated on the proper use of the device and understanding why it was useful at that specific stage of their rehabilitation. When physiotherapists contextualised the device's role in recovery and tailored the treatment to the individual's needs, participants reported greater acceptance of the device. This patient‐centred approach was crucial in building confidence and reducing device resistance.

Take it very gradually, providing explanations. Show the device, demonstrate how to use it, and explain what it’s for….—Participant 2, 27 yo, woman

#### 3.1.3 Theme 3: ‘A Compact Trainer for PFMT’

This theme was generated to encompass participants' insights on their overall experience with invasive devices, focussing on how these tools were used and contextualised within PFMT, emphasising their role after they gained confidence in using them (Table 6).

Participants reported that the devices' primary function was to enhance their awareness of their pelvic floor muscles. Specifically, they emphasised the role of visual and audio biofeedback cues in gradually helping them increase muscle awareness, ultimately fostering a sense of mastery over pelvic floor contraction.

The benefit is seeing—because it’s a biofeedback technique—seeing visually how my contraction was progressing and following it step by step. It motivated me to do it properly because I could see it happening—Participant 4, 69 yo, man

Thanks to the biofeedback function, participants found that using the devices was crucial in helping them become self‐managed over time. The flexibility and ease of use of the devices allowed participants to independently train their pelvic floor muscles while monitoring their progress session by session, fostering a sense of empowerment. One participant shared that having the device at home served as a tool for ‘refreshing’ on contracting her pelvic floor muscles.

I think having it available for a ‘refresh’ of the contraction perception would be ideal.—Participant 7, 53 yo, woman

Participants noted that the structured nature of the programmes offered by these devices helped address the adherence to PFMT, which was experienced as a primary challenge. The device offered clear guidance on timing and contractions (e.g., fast or endurance), reducing the mental effort required and encouraging more frequent training. This made the devices practical and compact ‘trainers’ for PFMT, allowing participants to use them anywhere and anytime.

The advantage is knowing exactly how long it takes. If the programme is 20 minutes, it’s 20 minutes, no more. So, it’s easy to plan for.—Participant 8, 36 yo, woman

However, participants stressed that these devices were merely supportive tools within the broader PFMT framework. Active engagement was essential to avoid passive use of the devices. While using them, participants needed to remain focused and actively contract their pelvic floor muscles to build awareness, which is required for the following stages of PFMT, requiring independence from the device.

I think being an active part of rehabilitation really makes a difference. It makes you feel less useless during these times.—Participant 2, 27 yo, woman

#### 3.1.4 Theme 4: “Users” Needs: Privacy, Accessibility, Usability, and Autonomy’

This last theme was generated to capture participants' perceived barriers faced when using invasive devices during PFMT and their suggestions for improvements (Table 7).

Participants highlighted the difficulty of creating an appropriate and private setting for using the device. For many, the need to undress, often lying down, and ensuring complete privacy at home was a source of anxiety. Additionally, the fear of being interrupted or discovered by relatives while using the device increased their discomfort and made consistent use challenging. Another barrier that hindered discretion was identified in the device design. Participants noted that larger, less portable devices were complex to bring, making them inconvenient to transport or use outside clinical settings.

You need a quiet place to do them, so it requires some preparation. If you’re feeling stressed, your mind races, and it could cause anxiety in certain individuals.—Participant 2, 27 yo, woman

An important barrier reported was the low accessibility of the devices in terms of costs, operator‐dependency and clear settings. Devices' costs were reported as expensive and often inaccessible for regular personal use. These constraints limited their use of supervised PFMT sessions in clinical settings, as patients did not have the knowledge and recourses to have them at home. Moreover, participants reported that certain devices required assistance or setup by physiotherapists, reducing their autonomy. Participants expressed a desire for simpler devices, with accessible user interfaces that the user could independently manage.

Some of these devices aren’t accessible because they can only be used by certain professionals. The ones you can use on your own don’t, in my opinion, provide the same benefits—Participant 2, 27 yo, woman

Practical challenges in adopting invasive devices also emerged, such as the necessity of lubricants, which participants felt were not sufficiently emphasised in device instructions but were crucial for comfort. One participant, who was blind, noted the lack of accessibility features, such as vocal assistance, which did not facilitate autonomous use.

Many people assume you don’t need lubricant, but I think this should be made clear. I read about it or was told, but the availability to adapt or make changes is crucial if someone isn’t ready yet—Participant 2, 27 yo, woman

To conclude, participants expressed the need for invasive devices to be more user‐friendly, discreet, and adaptable to diverse needs, enabling greater autonomy and integration into daily life while reducing the logistical and psychological barriers to their use.

Ease of use. I had trouble with some devices, for instance, setting the heat… It should be something that people can manage themselves, automatically.—Participant 2, 27 yo, woman

## 4 Discussion

This study explored the experiences of people with UI using invasive devices during PFMT. Participants shared their emotional responses upon being introduced to the devices, ranging from rejection to a sense of obligation and eventually acceptance (‘A Tool for Pelvic Floor Muscles: from Obligation to Acceptance’). Physiotherapists could support people doing PFMT in managing these emotions positively using strategies like gradual exposure, patient education and tailored rehabilitation process (‘Building Trust Through Gradual Exposure and Tailored Guidance’). As participants gained experience with the invasive devices, they recognised how biofeedback function helped them to increase their pelvic floor muscle awareness and self‐management skills, especially when actively engaged during the use (‘A Compact Trainer for PFMT’). Finally, participants expressed a desire for more practical, accessible and easy to use devices (‘”Users” Needs: Privacy, Accessibility, Usability, and Autonomy’).

Participants reported a sense of anxiety and discomfort about the idea of using endocavitary devices, perceiving them as ‘not natural’, in line with the results of another study on the overall PFMT experience (Giardulli et al. 2024). Specifically, a participant worried about ’what other people would think about me?’ a thought potentially linked to societal taboos surrounding the pelvic area, which potentially may affect engagement (Xu et al. 2023), as reported by other authors (Xu et al. 2023; Elenskaia et al. 2011; Pedofsky et al. 2021). However, not all participants shared this feeling, as some viewed devices as not an issue, especially if they had a positive relationship with their bodies. Men accepted to use anal devices as an obligation step to improve their symptoms, despite describing the experience as unpleasant. This could be linked to societal gendered norms (Connell and Messerschmidt 2005). Samulowitz A. et al. investigated gendered norms in chronic pain and reported that men, in line with hegemonic masculinity, associate men's gender identity with strength, power and stoicism (Samulowitz et al. 2018). In this context, hegemonic masculinity might have led participants to perceive the use of endocavitary devices as feminine and, therefore, a threat to their masculinity (Connell and Messerschmidt 2005). Clinicians should evaluate if the use of an endocavitary device is fundamental for men with UI, and if so, address gendered misconceptions through education, presenting the device as a supportive treatment rather than a threat to masculinity.

To overcome negative feelings, participants emphasised the crucial role of a trusting relationship and open communication with the physiotherapists. Being introduced to the invasive devices with kindness, discretion and a gradual approach made acceptance easier (Navarro‐Brazález et al. 2021). These results resonate with other studies highlighting how the role of the physiotherapist can influence feelings about PFMT (Giardulli et al. 2024; Hay‐Smith et al. 2015). Trust in health professionals was considered crucial as it helped participants view devices as valuable rehabilitation tools. Providing education about the devices, explaining their utility and purpose, and contextualising them within patients' needs further improved acceptance. In this sense, patient education is a well‐known strategy to increase patients' satisfaction with PFMT programmes (Leite et al. 2024; Grant and Currie 2020). These supportive strategies described by participants, such as education, reassurance, and progressive exposure, align with behavioural intervention functions such as persuasion and training, facilitating users' capability, opportunity, and motivation to empower PFMT users (Giardulli et al. 2025).

The biofeedback function of the invasive devices was highly valued for improving participants' ability to contract pelvic floor muscles, ultimately improving their self‐management skills, consistent with other studies (Xu et al. 2023; Perrier and Aumont 2024). Having a device available for home use allowed participants to engage in structured training independently while monitoring their progress. Similarly, Perrier and Aumont highlighted the benefits of using an endocavitary device for improving self‐management in women with UI (Perrier and Aumont 2024). However, participants emphasised how devices should be used with an active approach, focussing on how they contract pelvic floor muscles to become independent and progress within the PFMT without relying on devices. They stressed the importance of cognitive engagement during use to effectively learn pelvic floor muscle control, rather than treating devices as passive treatments. This reflects the view of PFMT as a behavioural therapy, where sustained improvements rely on active participation, motor learning, and psychological involvement, as highlighted by Frawley et al. (Frawley et al. 2017). Although quantitative data about the added value of devices to PFMT appears inconsistent regarding clinical symptom improvements (Fernandes et al. 2025; Benedetto et al. 2023), our findings suggest how devices could play a role in facilitating PFMT engagement based on patients' perceptions. Specifically, participants' narratives point to psychological benefits such as confidence, autonomy and a greater sense of control. More research is needed to understand which users are most likely to benefit from these behavioural effects, and to what extent.

Participants also identified some barriers to device use. Privacy concerns were significant, as training required creating an environment where they could undress and lie down without fear of interruption, consistent with findings from studies in other cultural contexts (Giardulli et al. 2024; Mishra et al. 2020). Some devices lacked discretion, making them unsuitable for travel or visible at home. Additionally, cost was a challenge for some participants, and the devices perceived as most effective often required professional setup or specialised licences, limiting accessibility. Finally, improving the accessibility of these devices for individuals with disabilities, such as those who are blind, is essential for future devices' development.

Based on participants' experience, possible alternatives could include the development of external, non‐invasive biofeedback devices, such as pressure sensors, that maintain the benefits of pelvic floor muscle awareness while avoiding the discomfort of endocavitary devices, though without offering physical stimulation (e.g., electrostimulation). Compact, wireless devices would allow a discreet home use, reducing anxiety and privacy concern while increasing autonomy. Audio instructions or tactile feedback features (e.g., vibration) could enhance accessibility for users with visual and hearing impairments, respectively. To support users with limited hand mobility or dexterity (e.g., due to arthritis or neurological conditions), devices should have intuitive controls, voice commands, or app‐based interfaces with accessibility features. For users with cognitive impairments, simplified instructions and guided, step‐by‐step feedback could facilitate safe and effective use (B. A. Lee et al. 2017). Devices usable in seated or standing positions would address physical and environmental limitations, making the training more adaptable to daily life. In line with participants' emphasis on active engagement, future designs could include interactive features that promote conscious control of muscle contractions, supporting motor learning rather than passive stimulation use. Finally, co‐designing such devices with users from diverse backgrounds—considering gender, body awareness, and usability preferences—may result in more acceptable, effective, and empowering tools for PFMT.

Some limitations need to be addressed. Participants in this study identified as heterosexual and cisgender, limiting the transferability of findings to LGBTQIA+populations and requiring further research. Additionally, our sample consisted of Italian patients with UI, potentially excluding perspectives from diverse cultural or ethnic backgrounds. Moreover, we included experiences with various types of invasive devices, provided they were endocavitary. Future research focussing on each device type individually could deepen our understanding of users' specific experiences. The study also included people willing to share their experiences, not considering those less inclined to discuss such topics. Moreover, online interviews could also have limited participation from people less comfortable with technology. Online interviews could have hindered the development of trust between participant and interviewer—while some participants may have felt more comfortable disclosing from the privacy of their home, others might have preferred the familiarity and warmth of face‐to‐face interaction. Finally, this study focused exclusively on participants' therapeutic experiences and did not explore potential sexual pleasure or uses beyond the clinical context, as this was outside its scope and aim. Future studies should consider broader populations to capture diverse perspectives, including how therapeutic devices may intersect with cultural or sexual meanings. Despite these limitations, the study offers valuable insights into the experiences of using invasive devices in people doing PFMT, highlighting critical factors influencing acceptance, usability, and self‐management outcomes.

This study highlights the emotional experiences of people with UI using endocavitary devices during PFMT. Participants initially experienced anxiety and discomfort, perhaps influenced by societal taboos. However, these feelings were mitigated through gradual exposure to the devices, positive communication, and education provided by physiotherapists. When used with an active participation, focussing on how to contract the pelvic floor muscles, the devices were particularly valued for enhancing pelvic floor muscle awareness and self‐management skills and fostering empowerment. Challenges, such as privacy concerns, cost, and usability, highlight the need for improvements in device design and accessibility. Finally, findings emphasise a supportive therapeutic relationship and tailored guidance in promoting device acceptance and PFMT engagement in UI management.

## 5 Implications of Physiotherapy Practice

Physiotherapists play a fundamental role in guiding patients through the emotional and practical challenges of using invasive devices during PFMT. By fostering trust, providing gradual exposure, and offering tailored education, physiotherapists can help patients overcome discomfort and societal stigmas, ultimately improving PFMT engagement. These findings also highlight the importance of active participation in PFMT, as biofeedback devices enhance pelvic floor muscle awareness and self‐management. Addressing barriers such as privacy, cost, and accessibility is crucial for optimising treatment adherence and patient empowerment.

## Author Contributions

All authors made substantial contributions to the conception or design of the work or the acquisition, analysis, or interpretation of data. All authors drafted the work or revised it critically for important intellectual content. All authors approved the version to be published. All authors agree to be accountable for all aspects of the work in ensuring that questions related to the accuracy or integrity of any part of the work are appropriately investigated and resolved.

## Ethics Statement

This study was conducted respecting the Declaration of Helsinki and reported following the Consolidated Criteria for Reporting Qualitative Research (COREQ). Ethical approval was obtained from the Ethics Committee for University Research (CERA), University of Genova (approval date: 31/10/2023; CERA2023/80). The participants read and signed the informed note and the informed consent before participating.

## Consent

The participants read and signed the informative note and the informed consent before participating.

## Conflicts of Interest

The authors declare no conflicts of interest.

## Permission to Reproduce Material From Other Sources

The authors have nothing to report.

## Data Availability

The data presented in this study are available within the study.

## References

[pri70071-bib-0001] Alouini, S. , S. Memic , and A. Couillandre . 2022. “Pelvic Floor Muscle Training for Urinary Incontinence With or Without Biofeedback or Electrostimulation in Women: A Systematic Review.” International Journal of Environmental Research and Public Health 19, no. 5: 2789. 10.3390/IJERPH19052789. 35270480 PMC8910078

[pri70071-bib-0002] Benedetto, G. , B. Simone , L. Gaia , J. Mirko , B. Ottavia , and T. Marco . 2023. “The Added Value of Devices to Pelvic Floor Muscle Training in Radical Post‐Prostatectomy Stress Urinary Incontinence: A Systematic Review With Metanalysis.” PLoS One 18, no. 9: e0289636. 10.1371/JOURNAL.PONE.0289636. 37768987 PMC10538711

[pri70071-bib-0003] Bo, K. , H. C. Frawley , B. T. Haylen , et al. 2017. “An International Urogynecological Association (IUGA)/International Continence Society (ICS) Joint Report on the Terminology for the Conservative and Nonpharmacological Management of Female Pelvic Floor Dysfunction.” Neurourology and Urodynamics 36, no. 2: 221–244. 10.1002/NAU.23107. 27918122

[pri70071-bib-0004] Booth, A. , K. Hannes , A. Harden , J. Noyes , J. Harris , and A. Tong . 2014. “COREQ (Consolidated Criteria for Reporting Qualitative Studies).” Guidelines for Reporting Health Research: A User’s Manual: 214–226. 10.1002/9781118715598.CH21.

[pri70071-bib-0005] Braun, V. , and V. Clarke . 2021a. Thematic Analysis: A Practical Guide.

[pri70071-bib-0006] Braun, V. , and V. Clarke . 2021b. “Can I Use TA? Should I Use TA? Should I Not Use TA? Comparing Reflexive Thematic Analysis and Other Pattern‐Based Qualitative Analytic Approaches.” Counselling and Psychotherapy Research 21, no. 1: 37–47. 10.1002/CAPR.12360.

[pri70071-bib-0007] Buckley, B. S. , and M. C. M. Lapitan . 2010. “Prevalence of Urinary Incontinence in Men, Women, and Children—Current Evidence: Findings of the Fourth International Consultation on Incontinence.” Urology 76, no. 2: 265–270. 10.1016/J.UROLOGY.2009.11.078. 20541241

[pri70071-bib-0008] Cacciari, L. P. , M. Morin , M. H. Mayrand , M. Tousignant , M. Abrahamowicz , and C. Dumoulin . 2021. “Pelvic Floor Morphometrical and Functional Changes Immediately After Pelvic Floor Muscle Training and at 1‐Year Follow‐Up, in Older Incontinent Women.” Neurourology and Urodynamics 40, no. 1: 245–255. 10.1002/NAU.24542. 33075192

[pri70071-bib-0009] Connell, R. W. , and J. W. Messerschmidt . 2005. “Hegemonic Masculinity: Rethinking the Concept.” Gender & Society 19, no. 6: 829–859. 10.1177/0891243205278639.

[pri70071-bib-0010] Corrado, B. , B. Giardulli , F. Polito , S. Aprea , M. Lanzano , and C. Dodaro . 2020. “The Impact of Urinary Incontinence on Quality of Life: A Cross‐Sectional Study in the Metropolitan City of Naples.” Geriatrics 5, no. 4: 1–14. 10.3390/GERIATRICS5040096. 33233663 PMC7709681

[pri70071-bib-0011] Elenskaia, K. , K. Haidvogel , C. Heidinger , D. Doerfler , W. Umek , and E. Hanzal . 2011. “The Greatest Taboo: Urinary Incontinence as a Source of Shame and Embarrassment.” Wiener Klinische Wochenschrift 123, no. 19–20: 607–610. 10.1007/S00508-011-0013-0. 21935649

[pri70071-bib-0012] Fernandes, A. C. N. , C. H. Jorge , M. Weatherall , I. V. Ribeiro , S. A. Wallace , and E. J. C. Hay‐Smith . 2025. “Pelvic Floor Muscle Training With Feedback or Biofeedback for Urinary Incontinence in Women.” Cochrane Database of Systematic Reviews 3. 10.1002/14651858.CD009252.PUB2. PMC11895424 40066950

[pri70071-bib-0013] Frawley, H. C. , S. G. Dean , S. C. Slade , and E. J. C. Hay‐Smith . 2017. “Is Pelvic‐Floor Muscle Training a Physical Therapy or a Behavioral Therapy? A Call to Name and Report the Physical, Cognitive, and Behavioral Elements.” Physical Therapy 97, no. 4: 425–437. 10.1093/PTJ/PZX006. 28499001

[pri70071-bib-0014] Giardulli, B. , G. Bertoni , I. Coppola , O. Buccarella , M. Testa , and S. Battista . 2025. “Pelvic Floor Muscle Training Strategies to Empower Patients: A Critical Incident Qualitative Study.” European Journal of Physiotherapy: 1–14. 10.1080/21679169.2025.2462329.

[pri70071-bib-0015] Giardulli, B. , I. Coppola , T. · Marco , O. Buccarella , and B. · Simone . 2024. “The Experience of Pelvic Floor Muscle Training in People With Urinary Incontinence: A Qualitative Study.” Sexuality and Disability: 1–19. 10.1007/S11195-024-09863-W.

[pri70071-bib-0016] Grant, A. , and S. Currie . 2020. “Qualitative Exploration of the Acceptability of a Postnatal Pelvic Floor Muscle Training Intervention to Prevent Urinary Incontinence.” BMC Women's Health 20: 1–8. 10.1186/S12905-019-0878-Z/PEER-REVIEW. 31952500 PMC6967084

[pri70071-bib-0017] Hay‐Smith, J. , S. Dean , K. Burgio , D. McClurg , H. Frawley , and C. Dumoulin . 2015. “Pelvic‐Floor‐Muscle‐Training Adherence ‘Modifiers’: A Review of Primary Qualitative Studies‐2011 ICS State‐of‐the‐Science Seminar Research Paper III of IV.” Neurourology and Urodynamics 34, no. 7: 622–631. 10.1002/NAU.22771. 25998067

[pri70071-bib-0018] Kondo, A. , Y. Yamada , and R. Niijima . 1995. “Treatment of Stress Incontinence by Vaginal Cones: Short‐ and Long‐Term Results and Predictive Parameters.” British Journal of Urology 76, no. 4: 464–466. 10.1111/J.1464-410X.1995.TB07746.X. 7551882

[pri70071-bib-0019] Lee, B. A. , S. J. Kim , D. K. Choi , O. Kwon , H. R. Na , and S. T. Cho . 2017. “Effects of Pelvic Floor Muscle Exercise on Urinary Incontinence in Elderly Women With Cognitive Impairment.” International Neurourology Journal 21, no. 4: 295–301. 10.5213/INJ.1734956.478. 29298469 PMC5756818

[pri70071-bib-0020] Lee, H. N. , S. Y. Lee , Y. S. Lee , J. Y. Han , M. S. Choo , and K. S. Lee . 2013. “Pelvic Floor Muscle Training Using an Extracorporeal Biofeedback Device for Female Stress Urinary Incontinence.” International Urogynecology Journal 24, no. 5: 831–838. 10.1007/S00192-012-1943-4. 23052631

[pri70071-bib-0021] Leite, A. M. C. , R. C. de Araújo , A. V. R. dos Santos , et al. 2024. “Efficacy of Educational Instructions on Pelvic Floor Muscle Training in the Treatment of Urinary Incontinence: Systematic Review and Meta‐Analysis.” Neurourology and Urodynamics 43: 219–235. 10.1002/NAU.25287. 37712496

[pri70071-bib-0022] Lim, R. , M. L. Liong , W. S. Leong , N. A. K. Khan , and K. H. Yuen . 2016. “Effect of Stress Urinary Incontinence on the Sexual Function of Couples and the Quality of Life of Patients.” Journal d'Urologie 196, no. 1: 153–158. 10.1016/J.JURO.2016.01.090. 26812304

[pri70071-bib-0023] Maher, L. , and G. Dertadian . 2018. “Qualitative Research.” Addiction 113, no. 1: 167–172. 10.1111/ADD.13931. 28786222

[pri70071-bib-0024] Malterud, K. , V. Dirk Siersma , and A. D. Guassora . 2016. “Sample Size in Qualitative Interview Studies: Guided by Information Power.” Qualitative Research Health 26, no. 13: 1753–1760. 10.1177/1049732315617444. 26613970

[pri70071-bib-0025] Mazur‐Bialy, A. I. , D. Kołomańska‐Bogucka , C. Nowakowski , and S. Tim . 2020. “Urinary Incontinence in Women: Modern Methods of Physiotherapy as a Support for Surgical Treatment or Independent Therapy.” Journal of Clinical Medicine 9: 1211. 10.3390/JCM9041211. 32340194 PMC7230757

[pri70071-bib-0026] Mishra, G. D. , D. Kumar , G. A. Pathak , and B. S. Vaishnav . 2020. “Challenges Encountered in Community‐Based Physiotherapy Interventions for Urinary Incontinence Among Women in Rural Areas of Anand District of Gujarat, India.” Indian Journal of Public Health 64, no. 1: 17–21. 10.4103/IJPH.IJPH_436_18. 32189677

[pri70071-bib-0027] National Institute for Health and Care Excellence (NICE) . 2019. Urinary Incontinence and Pelvic Organ Prolapse in Women: Management NICE Guideline. 31211537

[pri70071-bib-0028] Navarro‐Brazález, B. , F. Vergara‐Pérez , V. Prieto‐Gómez , B. Sánchez‐Sánchez , M. J. Yuste‐Sánchez , and M. Torres‐Lacomba . 2021. “What Influences Women to Adhere to Pelvic Floor Exercises After Physiotherapy Treatment? A Qualitative Study for Individualized Pelvic Health Care.” Journal of Personalized Medicine 11, no. 12: 1368. 10.3390/JPM11121368. 34945840 PMC8706048

[pri70071-bib-0029] Oben, P. 2020. “Understanding the Patient Experience: A Conceptual Framework.” Journal of Patient Experience 7, no. 6: 906–910. 10.1177/2374373520951672. 33457518 PMC7786717

[pri70071-bib-0030] Ong, T. A. , S. Y. Khong , K. L. Ng , et al. 2015. “Using the Vibrance Kegel Device With Pelvic Floor Muscle Exercise for Stress Urinary Incontinence: A Randomized Controlled Pilot Study.” Urology 86, no. 3: 487–491. 10.1016/J.UROLOGY.2015.06.022. 26142713

[pri70071-bib-0031] Pedofsky, L. , P. M. F. Nielsen , D. Budgett , K. Nemec , C. Dumoulin , and J. Kruger . 2021. “Using Codesign to Develop a Mobile Application for Pelvic Floor Muscle Training With an Intravaginal Device (Femfit®).” Neurourology and Urodynamics 40, no. 8: 1900–1907. 10.1002/NAU.24775. 34464005

[pri70071-bib-0032] Perrier, E. T. , and L. Aumont . 2024. “Pelvic Floor Muscle Training Using the Perifit Device for the Treatment of Urinary Incontinence: A Pragmatic Trial Using Real‐World Data.” Womens Health Rep 5, no. 1: 250–258. 10.1089/WHR.2023.0172. PMC10956527 38516650

[pri70071-bib-0033] Pizzol, D. , J. Demurtas , S. Celotto , et al. 2021. “Urinary Incontinence and Quality of Life: A Systematic Review and Meta‐Analysis.” Aging‐Clinical & Experimental Research 33, no. 1: 25–35. 10.1007/S40520-020-01712-Y. 32964401 PMC7897623

[pri70071-bib-0034] Samulowitz, A. , I. Gremyr , E. Eriksson , and G. Hensing . 2018. “‘Brave Men’ and ‘Emotional Women’: A Theory‐Guided Literature Review on Gender Bias in Health Care and Gendered Norms Towards Patients With Chronic Pain.” Pain Research and Management 2018: 6358624–6358714. 10.1155/2018/6358624. 29682130 PMC5845507

[pri70071-bib-0035] Società Italiana di Urologia, European Association of Urology . 2019. Linee Guida EAU sull’Incontinenza Urinaria Negli Adulti. Collane Linee Guida.

[pri70071-bib-0036] Thomas, H. S. , A. W. Lee , B. Nabavizadeh , et al. 2021. “Evaluating the Primary Use, Strengths and Weaknesses of Pelvic Floor Muscle Training Devices Available Online.” Neurourology and Urodynamics 40, no. 1: 310–318. 10.1002/NAU.24560. 33137215

[pri70071-bib-0037] (UK) NGA . 2021. Pelvic Floor Muscle Training for the Prevention of Pelvic Floor Dysfunction. Pelvic Floor Muscle Training for the Prevention of Pelvic Floor Dysfunction: Pelvic Floor Dysfunction: Prevention and Non‐Surgical Management: Evidence Review F.

[pri70071-bib-0038] Wang, L. , Y. Li , Z. Qi , and W. Wang . 2023. “Barriers and Facilitators of the Implementation of the Application of Pelvic Floor Muscle Training in Patients With Prostate Cancer: A Scoping Review.” Frontiers in Public Health 11. 10.3389/FPUBH.2023.1191508/PDF. PMC10523151 37771836

[pri70071-bib-0039] Xu, P. , Y. Jin , P. Guo , et al. 2023. “Barriers and Enablers of Pelvic Floor Rehabilitation Behaviours in Pregnant Women With Stress Urinary Incontinence: A Qualitative Analysis Using the Theoretical Domains Framework.” BMC Pregnancy and Childbirth 23, no. 1: 300. 10.1186/S12884-023-05633-2. 37118702 PMC10148524

[pri70071-bib-0040] Yunus, N. A. , T. olde Hartman , P. Lucassen , et al. 2022. “Reporting of the Translation Process in Qualitative Health Research: A Neglected Importance.” International Journal of Qualitative Methods 21. 10.1177/16094069221145282.

